# Experiences in Emergency Cardiac Surgery of COVID-19-Positive Patients: A Case Series

**DOI:** 10.7759/cureus.42799

**Published:** 2023-08-01

**Authors:** Takanori Hishikawa, Takeki Ohashi, Soichiro Kageyama, Akinori Kojima

**Affiliations:** 1 Cardiac Surgery, Nagoya Tokushukai General Hospital, Kasugai, JPN

**Keywords:** emerging infectious disease, emergency surgery, cardiac surgery, covid-19, nosocomial infections

## Abstract

The coronavirus disease-2019 (COVID-19) pandemic has placed many restrictions on medical care. The timing of surgical treatment has been particularly affected, with patients experiencing delayed operation dates. This report describes three patients with preoperative COVID-19-positive diagnoses, tested with reverse transcription-polymerase chain reaction, who were urgently treated surgically with excellent results. Case 1 involved an 89-year-old woman with a left ventricular rupture after a myocardial infarction. Case 2 involved a 52-year-old male patient with an acute type A aortic dissection. Case 3 involved a patient with an occlusion of an autologous dialysis shunt. All patient conditions were either life-threatening or overwhelmed hospital beds without surgical treatment. In Japan, we still experience cases where surgery is refused because of COVID-19 positivity, even if emergency surgery is necessary. This report describes three cases where standard precautions were taken, postoperative management was devised, and good results were achieved.

## Introduction

The coronavirus disease 2019 (COVID-19) has had a major impact on daily medical treatment. To prevent nosocomial infections and other organ complications, it is recommended that patients who test positive wait at least seven weeks before surgery [1-3]. However, in cardiovascular surgery, some patients may die if an urgent or emergency procedure is delayed to make it an elective procedure. We believe there is controversy among regions and countries regarding what to do in emergency cases of patients with COVID-19 detected using reverse transcription polymerase chain reaction (RT-PCR). Here, we report three COVID-19-positive patients who underwent surgical treatment of the heart, aortic, and peripheral blood vessel regions with favorable results.

## Case presentation

Case 1

An 89-year-old female living in a nursing home decreased her food intake for approximately a week. Typically, the patient was independent in her activities of daily living. When a carer visited her room during dinner care, the patient was found unconscious and was brought to our hospital for emergency care. The initial vital signs showed a BP of 83/64 mmHg, a pulse of 116 beats/minute, a temperature of 36.5℃, and an oxygen saturation of 88% with a reservoir mask. While in the emergency room, a computed tomography (CT) scan of the head was performed to investigate the cause of the impaired consciousness, but no cause was found.

**Figure 1:**
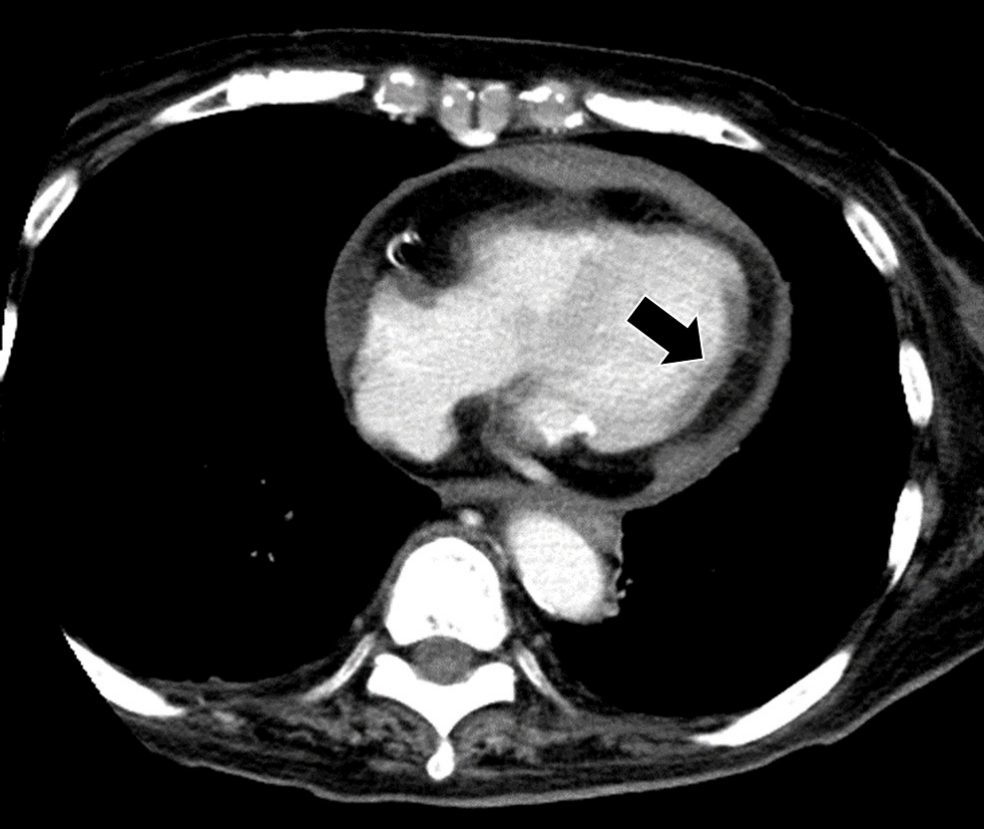
Cardiac contrast CT (Axial) The results of coronary angiography CT showed a low-absorption area with poor contrast in the posterior wall region.

**Figure 2:**
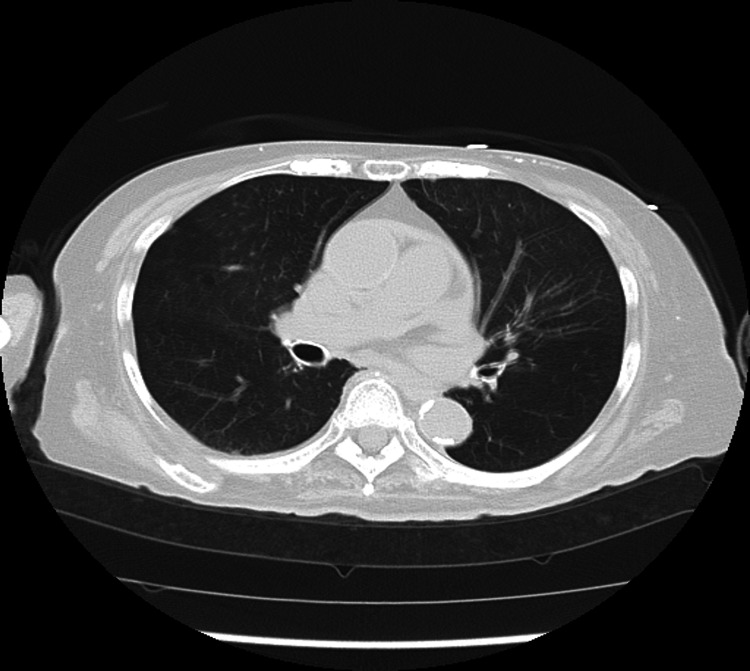
The lung field CT image of a COVID-19-positive patient (Case 1). There was no obvious image of pneumonia in the lung field.

Conversely, a chest CT showed that this patient had a left ventricular rupture due to myocardial infarction (Figure 1). This patient was diagnosed with impaired consciousness due to low output syndrome caused by the left ventricular rupture. The thoracoabdominal CT showed no obvious consolidation indicative of pneumonia (Figure 2). However, all patients must undergo RT-PCR before emergency surgery to prevent nosocomial infections. Routine RT-PCR before surgery was positive for COVID-19. Her bloody pericardial fluid had increased over time when compared to her arrival. Although a vasopressor was used in the emergency room, blood pressure maintenance was difficult, and we decided that we needed to perform life-saving emergency surgery. Standard precautions using appropriate personal protective equipment consistently by all personnel entering the patient's room were taken to avoid contact with the patient's bodily fluids, and the patient was intubated in the isolation emergency room. Surgery was performed in a negative air pressure isolation room to contain the virus. After opening the chest cavity, we observed a ruptured site in the posterior wall area sutured with 3-0 SURGIPRO®; hemostasis was achieved. The patient was admitted postoperatively to the intensive care unit (ICU). She was isolated in a negative-pressure room, and standard infection control measures were taken before entering and exiting the ICU. In-room communication with the staff was minimized using radio equipment. Six to seven days after testing positive, the patient's RT-PCR was negative, allowing extubation. The patient was transferred to a rehabilitation center on the fifteenth postoperative day. No nosocomial infections were identified in this case.

Case 2

**Figure 3:**
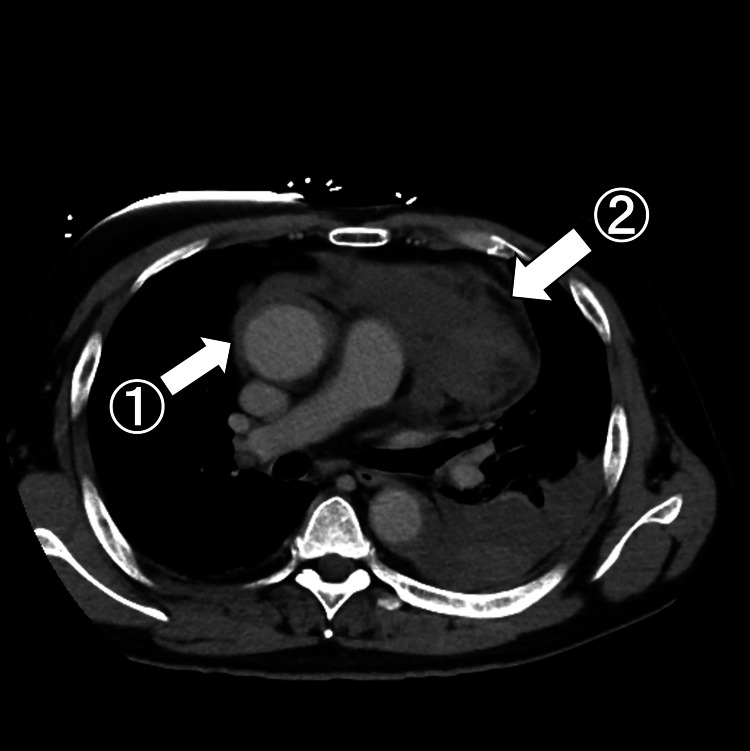
Chest contrast CT Aortic dissection of Stanford type A is seen ①. After sternal compression, a large amount of blood is seen in the anterior aspect of the pulmonary artery ②.

**Figure 4:**
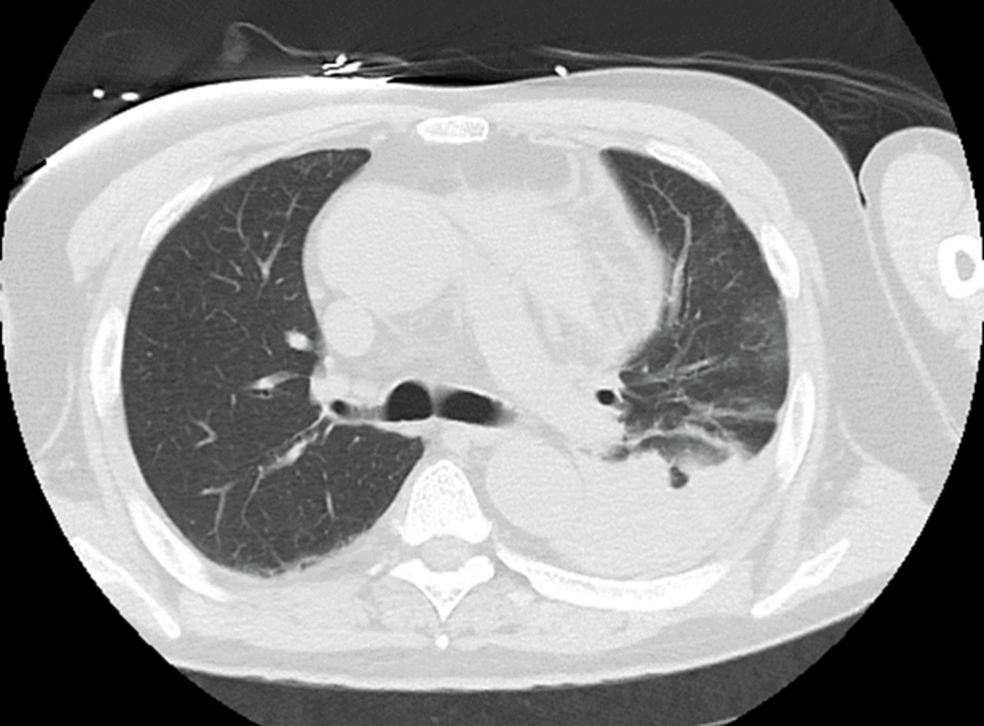
The lung field CT image of a COVID-19-positive patient (Case 2). There is no obvious infiltrative shadow in the lung field, but a hematogenous pleural effusion associated with rupture is observed in the left thoracic cavity.

A 52-year-old male patient was brought to the emergency room complaining of early-morning chest pain. During the ambulance transport, he lost consciousness once, and simultaneously, his carotid artery could not be palpated. The ECG waveform showed pulseless electrical activity and chest compressions were performed based on the determination of cardiac arrest. Spontaneous circulation was returned after one cycle of advanced cardiovascular life support with adrenaline. No tracheal intubation was performed during this time. On arrival at the hospital, the initial vital signs showed a BP of 65/46 mmHg, a pulse of 125 beats/minute, a temperature of 36.6℃, and an oxygen saturation of 99% with 4 L of nasal oxygen. The contrast-enhanced CT showed a Stanford type A acute aortic dissection with rupture and pericardial effusion (Figure 3). No obvious pneumonia was observed in the lung field (Figure 4). However, the patient was required to undergo RT-PCR prior to emergency surgery to prevent nosocomial infections and was found to be positive for COVID-19. This patient was vaccinated, and he had no prior contact with COVID-19-positive patients. On examination, we found a rupture of the ascending aorta and left haemothorax and determined that emergency surgery was necessary to save the patient's life. As in Case 1, the patient underwent surgical treatment in a negative-pressure room. He underwent surgical replacement of the ascending aorta with Triplex 24 mm®. The patient was placed postoperatively in a negative-pressure room in the ICU and was scheduled to be extubated after testing negative for COVID-19 on the sixth postoperative day. However, the patient self-extubated on the fifth postoperative day. There were no cerebral complications, and the patient was discharged on postoperative day 22. No nosocomial infections were observed in this patient.

Case 3

A 59-year-old female patient was undergoing maintenance dialysis at our hospital. She had an obstruction of the autologous intravascular shunt in her right forearm, and dialysis could not be performed. Her vital signs showed a BP of 125/72 mmHg, a pulse of 75 beats/minute, a temperature of 37.2℃, and an oxygen saturation of 99% in room air. While the COVID-19 RT-PCR test was positive, she had no fever or respiratory symptoms. Shunt ultrasonography showed poor blood flow on the venous side, and the patient was difficult to dialyze due to poor blood flow. Catheter-based percutaneous transluminal angioplasty was performed, and the patient could return home on a day-care basis without hospitalization. No nosocomial infections were observed in this patient.

## Discussion

COVID-19 infection has been reported to impact the surgical treatment of patients negatively. Glasbey et al. reported that 'patients operated within six weeks of SARS-CoV-2 diagnosis were at an increased risk of 30-day postoperative mortality and 30-day postoperative pulmonary complications' [1-3]. However, some diseases preclude patients from undergoing standby surgery. A COVID-19 RT-PCR test is now a prerequisite in our hospital before entering the operating room, even for emergency surgery. It has been confirmed that preoperative waiting time for RT-PCR does not adversely affect postoperative outcomes in patients with acute type A aortic dissection when the appropriate antihypertensive control is maintained [4]. However, the pathogenesis of COVID-19 is complex and includes activation of a systemic inflammatory cascade and accelerated thrombogenesis, resulting in systemic organ damage and a highly variable clinical course [5]. Cardiac surgery with extracorporeal circulation can cause similar pathologies, acting synergistically with the COVID-19 pathogenesis to cause excessive systemic inflammatory responses and alterations in the coagulation system, thereby increasing the potential for various postoperative complications. Early experiences with COVID-19-positive patients undergoing cardiac surgery reported high mortality and morbidity rates [6,7]. However, the few reports in the literature make it difficult to draw reasonable conclusions about the prognosis and outcome of patients suffering from cardiac surgery and COVID-19 and establish clear recommendations for their management. However, despite these limited reports, delayed postoperative respiratory failure requiring reintubation or tracheostomy appears to be a frequent complication in the immediate postoperative period. In addition, complications resulting from changes in the coagulation system (excessive bleeding and thromboprophylaxis) have been reported [8,9]. Postoperative hemostasis is also an important factor, especially in the case of major cardiac vascular surgery.

In some cases, excessive bleeding may require massive blood transfusions. Regarding prevention, we wore N95 particulate respirator-type masks when operating on COVID-19-positive patients in a negative-pressure room with anesthesia machines and covered the operating room walls in plastic. In addition, the number of supplies (needles, gauze, etc.) allocated to the operating room was limited compared with conventional circumstances. Further, radio communication was used to request any additional supplies when necessary. The door to the negative-pressure room was opened and closed as minimally as possible. Aerosol and droplet infection of patients with the novel coronavirus may pose a significant risk to surgeons; thus, to avoid risks during intubation and extubation of COVID-19-positive patients, surgeons and other medical personnel not involved in intubation of patients with or suspected of having the novel coronavirus infection were asked to remain outside the operating room until induction of anesthesia and completion of intubation and awakening from anesthesia and extubation. When leaving the room, all items that had come into contact with the infected patients were discarded and sealed. Contact with other patients was eliminated when entering and leaving the operating room, and staff members who let patients out of the operating room and those who returned to the ICU after leaving the operating room were changed. The operating room staff disinfected their hands before exiting the room and promptly took showers. The ICU nurses monitored the patients 24h a day using a remote camera. As a result, no nosocomial infections were observed in these three cases.

## Conclusions

This study describes three cases of COVID-19-positive patients who underwent emergency surgery with good results. Since COVID-19 increases the risk of prolonged tracheal intubation, if waiting time does not pose a risk to elective patients, a surgical waiting period of seven weeks or longer is desirable in patients with COVID-19 infection. However, in cases where a waiting period is not possible, the risk of nosocomial infection may be minimized when the necessary infection control measures are taken. Although currently, patients are refused emergency surgery because of COVID-19 infection and are therefore referred to our hospital, COVID-19 infection should not be a reason to avoid emergency surgery.
